# Implementation of AI in radiology: the perspective of referring physicians

**DOI:** 10.1186/s13244-025-02120-4

**Published:** 2025-10-31

**Authors:** Jennifer Gotta, Leon D. Grünewald, Vitali Koch, Scherwin Mahmoudi, Simon Bernatz, Elena Höhne, Teodora Biciusca, Aynur Gökduman, Christian Wolfram, Christian Booz, Jan-Erik Scholtz, Simon Martin, Katrin Eichler, Tatjana Gruber-Rouh, Andreas Bucher, Ibrahim Yel, Thomas J. Vogl, Philipp Reschke

**Affiliations:** Goethe University Hospital Frankfurt, Frankfurt am Main, Germany

**Keywords:** Artificial intelligence, Radiology, Physician’s attitudes, Data privacy

## Abstract

**Objectives:**

AI offers considerable potential to improve diagnostic accuracy and efficiency in radiology. However, its successful implementation depends largely on the trust and acceptance of referring physicians. This study examines physicians’ attitudes toward AI in radiology, identifying key facilitators and barriers to its clinical integration.

**Materials and methods:**

A total of 169 licensed physicians in Germany, including surgeons, internists, and general practitioners who frequently refer patients to radiology, were surveyed. Participants were recruited via a systematic review of hospital and practice websites. A structured online questionnaire assessed perceptions of AI, focusing on trust-related factors, preferred applications, and adoption barriers. Statistical analysis was conducted using R and Python.

**Results:**

Overall, 60% of respondents evaluated AI positively for enhancing diagnostic accuracy (mean score 3.7 ± 1.2). The most influential trust factor was model transparency (56.3%), followed by legal clarity on liability (25.0%) and strong data protection (11.7%). Transparency was rated significantly higher than other factors (*p* < 0.001). Preferred AI applications included lesion detection, research data analysis, and workflow management. Barriers to adoption included the “black box” nature of AI, unclear accountability, and data privacy concerns. Subgroup analysis revealed no significant variation in trust factors between specialties (*p* = 0.21).

**Conclusion:**

Physicians see AI as a promising tool in radiology but emphasize the need for greater transparency, clear legal responsibility, and secure data handling. Addressing these concerns through explainable AI models, legal frameworks, and robust data protection measures is essential for fostering trust and facilitating successful AI integration in clinical practice.

**Critical relevance statement:**

Understanding physicians’ concerns about AI transparency, liability, and data privacy is essential. Addressing these barriers is critical to ensuring responsible implementation, building trust, and enabling effective integration of AI into clinical radiology workflows.

**Key Points:**

- AI acceptance in radiology faces transparency and liability concerns.
- Lesion detection and data analysis were rated most beneficial by physicians.
- Clear regulation and explainability are key for clinical AI trust.

**Graphical Abstract:**

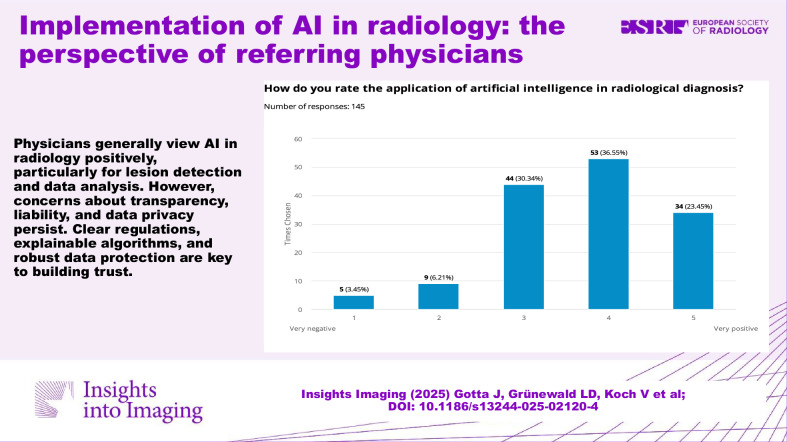

## Introduction

The field of diagnostic imaging has expanded significantly in recent years due to a surge in healthcare data. In the USA, for example, the frequency of computed tomography (CT) scans increased in number from 1990 with 13 million CT-scans annually, up to 62 million CT-scans in 2006 [1].

Artificial Intelligence (AI) has emerged as a transformative technology in the field of radiology, offering significant potential to enhance diagnostic accuracy, efficiency, and workflow optimization. The use of AI assists in identifying more anomalies by leveraging pattern recognition. AI can serve as a valuable tool, complementing the expertise of both novice and experienced radiologists in reducing the reporting time and increasing the share of correctly classified scans [2]. The phenomenon known as “satisfaction of search” describes instances where radiologists may miss additional pathologies after identifying an initial finding [3]. The use of AI as a supporting tool could contribute to detecting these missed lesions. In particular, a study suggests that an AI model can detect pulmonary nodules more accurately than six experienced radiologists [4, 5]. In emergency radiology, AI can immediately identify critical cases, such as strokes or pulmonary embolisms, and prioritize them to ensure the fastest possible treatment [6]. A less widely known but equally important goal for AI is reducing radiation exposure. Deep learning post-processing algorithms enable high image quality with lower radiation doses [7]. Additionally, AI algorithms provide an objective and consistent method for assessing changes in tumor size [8–10]. Given that CT volumetry of liver volume is time-consuming, AI algorithms can streamline the process and enhance clinical applications. Liver volume measurement is clinically significant for transplants, resections and evaluating liver fibrosis [11].

Despite its advantages, the integration of AI in clinical radiology faces substantial challenges, particularly related to trust and acceptance among referring physicians. Studies indicate that while many clinicians recognize the potential benefits of AI, there are concerns regarding the reliability and interpretability of AI-driven diagnostics, particularly in cases where human oversight may be limited [1, 12]. In radiology, this challenge is often referred to as the “black box” problem, where the underlying reasoning behind an AI model’s decisions is, in most cases of machine learning, opaque and not easily interpretable by humans [13, 14]. Trust remains a pivotal factor, especially as AI systems continue to evolve and demonstrate varying levels of accuracy across different imaging modalities [15].

The integration of AI in clinical routines has sparked considerable debate across various medical specialties. However, a significant research gap exists in evaluating these differing opinions. Therefore, this study aims to gain a deeper understanding of the perspectives of referring physicians towards the adoption of AI in radiological diagnosis, ultimately identifying key barriers and facilitators for the successful integration of AI in radiology.

## Materials and methods

### Participant selection

This prospective study was approved by the Institutional Review Board of the University Hospital Frankfurt and focused on licensed medical professionals in Germany, specifically surgeons, internists, and general practitioners, who frequently utilize radiological services in their practice. Eligibility required respondents to provide demographic information and answer at least one primary survey question. Exclusion criteria included retired physicians, those who rarely request radiological evaluations in their routine practice, and individuals who did not complete both the demographic section and any key survey questions.

Participants were identified through a systematic review of publicly accessible hospital and private clinic websites across Germany, ensuring a balanced representation from diverse institutions in urban and rural settings. To ensure nationwide representativeness, participant recruitment was stratified across all 16 federal states of Germany, encompassing both metropolitan areas and rural regions. A stratified random sampling strategy was employed to achieve a balanced distribution across different medical subspecialties and geographic regions.

In total, 2195 physician contacts were screened. From these, 453 were randomly selected and invited to participate, following stratification criteria that reflected the proportional distribution of medical specialties and regional coverage in Germany.

**Fig. 1:**
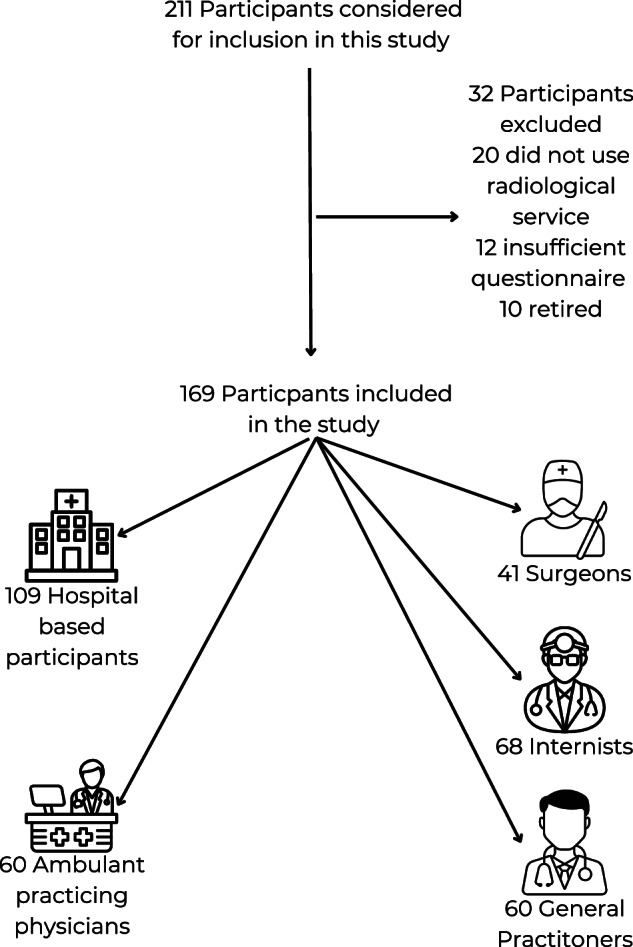
Flow chart of participant inclusion

Physicians from various career stages, including residents, specialists, senior consultants, and department heads, were included. Email addresses were collected from publicly available sources on institutional and practice websites. Eligible candidates were randomly selected and invited to participate via email. The invitation outlined the purpose of the study, highlighted the anonymous and voluntary nature of the survey, and provided a link to the online questionnaire. Prior to participation, all respondents provided informed consent. Ethical approval was not required, as the survey solely gathered physicians’ opinions. The study followed ethical guidelines, and all analyses complied with local data protection regulations. The study design is shown in Fig. 1.

### Questionnaire

A structured online survey was used to evaluate physician perceptions regarding the integration of AI in radiology. The questionnaire was specifically designed to examine three critical dimensions: (1) factors influencing trust in AI systems, (2) preferred clinical applications of AI, and (3) perceived barriers to adoption.

The questionnaire was custom-designed and included multiple-choice items, single-choice questions, Likert-scale ratings (ranging from 1 = strongly negative to 5 =  strongly positive), and open-ended queries. For comparative analysis, responses were stratified according to practice setting (private practice versus hospital-based) and medical specialty, with particular attention to differences between general practitioners, surgeons, and internists. This stratification enabled us to identify specialty-specific patterns in AI perception. To encourage participation despite the demanding workloads of physicians, the questionnaire was deliberately kept concise and time-efficient. This approach aimed to lower the threshold for participation and reduce potential barriers to completion (Fig. S1).

### Statistical analysis

The data were analyzed using R software (R Foundation for Statistical Computing; Version 2023.06.0 + 421) and Python (Version 3.12.6).

Descriptive statistics, including measures such as mean, median, mode, and standard deviation, were utilized to summarize the characteristics of the sample. To examine group differences, various statistical tests were applied, including *t*-tests, Mann–Whitney *U*-tests, Chi-square tests, and Kruskal–Wallis tests, with statistical significance defined as *p* < 0.05. For ranking questions, weighted scores were assigned according to the position of each item within the ranking, providing a clearer interpretation of the participants’ priorities. This method enabled a more nuanced understanding of how different factors were prioritized by the respondents.

## Results

### Overview of participant selection and characteristics

Of the initial pool of 211 physicians identified for the study, certain exclusions were applied for various reasons: 20 physicians did not routinely utilize radiology services, 12 only filled out the demographic section of the survey without answering any core questions, and 10 were found to be retired. Ultimately, 169 participants met the inclusion criteria: 68 internists, 41 surgeons, and 60 general practitioners. This corresponds to a completion rate of approximately 37% based on the invited sample (169/453). All general practitioners were based in private practices, whereas the internists and surgeons were hospital-based. Among the 109 hospital physicians, the breakdown included 87 assistant physicians, 20 senior physicians, and 1 chief physician (Fig. 1 and Table 1).

**Table 1:** Specialties and experience of the participants

	Times chosen	Percentage	Average experience in years	Standard deviation in years
Internists	68	40.23%	6.9	6.4
Surgeons	41	24.26%	11.5	11.24
General Practitioners	60	35.50%	19.2	10.6

### Participant demographics

After filtering out outliers, the average survey completion time was recorded at 10 min. The respondents had an average of 9.8 years of professional experience (Median: 5 years, Mode: 3 years), indicating that most participants were relatively early in their careers, with experience ranging from less than a year to 42 years. On average, internists had 6.9 years of experience (Median: 3 years), surgeons had 11.5 years (Median: 6 years), while general practitioners had notably more experience, averaging 19.2 years (Median: 18.5 years, *p* < 0.05).

### Physicians’ perceptions of AI in radiological diagnosis

**Fig. 2:**
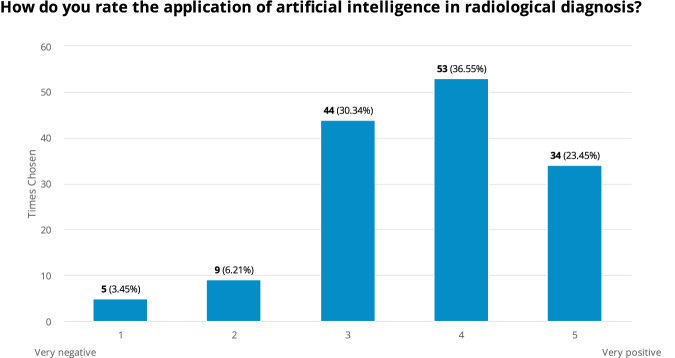
Physicians’ perceptions of AI in radiological diagnosis

The survey revealed a generally positive perception among physicians regarding the use of AI in radiological diagnostics. Out of 145 respondents, the majority rated AI favorably, with 36.6% selecting a score of 4 (moderately positive) and 23.5% rating it as 5 (very positive). The mean rating was 3.7 ± 1 (on a scale of 1 to 5), indicating a generally positive outlook but with some variability in opinions. Notably, a smaller proportion of respondents (9.7%) expressed negative views, rating AI as either 1 (very negative) or 2, regarding the use of AI in radiological diagnostics. Meanwhile, 30.3% of respondents rated AI as a 3, suggesting a neutral or slightly positive stance, indicating potential reservations or a cautious optimism regarding its application. No significant differences were observed among the surveyed specialties, with an average rating of 3.6 ± 1 (*p* = 0.12) (Fig. 2).

### Factors influencing trust in AI for radiological diagnostics

The majority (56.3%) emphasized the importance of transparency of AI models, particularly the need for explainability of how these systems work and full disclosure of the datasets used for training In addition, responsibility and liability emerged as a significant concern, with 25% of respondents highlighting the need for clearer guidelines on who bears responsibility in the event of diagnostic errors or adverse outcomes, whether it is the physician or the AI provider. Furthermore, data protection audits were identified by 11.7% of participants as a critical factor for building trust. A smaller portion (7%) of respondents indicated other factors, suggesting that while transparency, liability, and data protection are primary concerns, additional context-specific issues may also influence trust in AI (*p* < 0.001).

### Prioritization of AI application areas in radiology

Physicians were also asked to rate the importance of different AI applications in radiology, with rankings from 1 (highest priority) to 6 (lowest priority). The highest priority was given to lesion detection, which received a total score of 254, ranking it as a critical area.

**Fig. 3:**
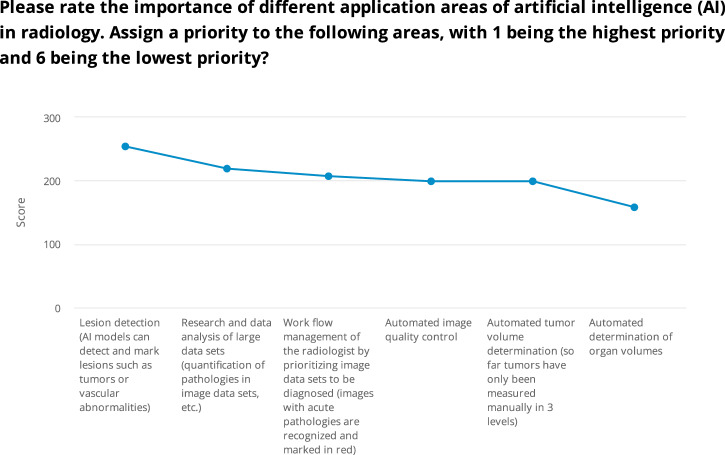
Prioritization of AI application areas in radiology

Research and data analysis of large datasets, including the quantification of pathologies, was rated as the second most important application, achieving a score of 219. In third place was workflow management, which focuses on prioritizing image datasets, especially those with an acute pathologies score of 207. Automated image quality control and automated tumor volume determination were closely ranked, each with a score of 199. The lowest priority was assigned to automated determination of organ volumes, which received a score of 158 from 60 participants (Fig. 3).

When analyzing the responses from different groups, surgeons, internists, and medical students consistently ranked lesion detection as the top priority. Additionally, internists highlighted automated tumor volume determination as another key area, giving it equal priority to lesion detection for preferred AI applications. These differences were highly statistically significant (*p* < 0.001), indicating that physicians assign distinct levels of importance to different AI applications in radiology.

## Discussion

AI has become an integral part of modern healthcare, particularly in medical and radiological advancements. This study provides valuable insights into physicians’ perspectives on AI in radiology. The key finding is that lesion detection emerged as the top-priority AI application, receiving strong support across all respondent groups.

Physicians viewed AI in radiology positively overall, with a mean rating of 3.7 ± 1, indicating general openness to its integration into clinical workflows despite some variability in opinions. Additionally, transparency was highlighted as the most critical factor for building trust in AI systems, with 56.3% of respondents emphasizing the importance of explainability and full disclosure of training datasets to ensure reliable and informed use of these tools in medical practice.

Most physicians in our study are optimistic about the integration of AI in radiological diagnostics, with over 60% rating it positively. Recent studies also showed that clinicians recognize AI’s potential to improve diagnostic accuracy. However, concerns about reliability and transparency hinder full acceptance [16] [17–19].

Overall, while AI is generally perceived positively, notable concerns remain regarding transparency, liability, and data protection. Transparency, identified by 56.3% of respondents as an important factor for building trust, underscores the importance of ensuring explainability in AI systems. This is consistent with the broader literature emphasizing that AI tools should not function as “black boxes” if they are to be trusted in clinical decision-making [13, 14, 20]. A quarter of our respondents highlighted responsibility and liability as a major concern, particularly regarding who would be accountable for diagnostic errors. Previous studies also identified the absence of clear legal frameworks for AI in healthcare as a substantial barrier to its widespread adoption [21]. Physicians fear that they may be held responsible for errors made by AI systems, which creates hesitancy in relying on such tools for critical diagnostic decisions [22–26].

Although data protection was not the top concern, with only 11.72% of participants selecting it as a priority, it remains an important consideration. In this context, recent studies have shown that while clinicians are generally supportive of AI, they emphasize the need for robust data protection protocols to ensure patient confidentiality. [27–31]. The findings from this study underscore the need for targeted strategies to improve trust in AI among physicians. Addressing transparency and liability concerns, as well as ensuring robust data protection, are essential steps to fostering confidence in AI systems. Additionally, focusing on AI applications that directly impact diagnostic accuracy, such as lesion detection and workflow optimization, may accelerate the acceptance of AI technologies in clinical settings.

Despite providing valuable insights into physicians’ perspectives on AI in radiology, several limitations must be acknowledged. Firstly, the focus on German physicians, may limit the generalizability of the findings to other healthcare systems or cultural contexts. Secondly, the reliance on self-reported data may introduce response bias, as participants might provide socially desirable answers or omit critical information. Thirdly, the survey’s use of predefined response options may have constrained the participants’ ability to fully express nuanced views, potentially affecting the depth of the data collected. Fourthly, the study included a large proportion of assistant physicians. These early-career professionals may have different experiences and expectations regarding AI compared to more senior physicians. Furthermore, the study focused primarily on internists, surgeons, and general practitioners, which may not fully capture the perspectives of other specialties, such as dermatologists or pediatricians, who also engage with radiological services.

The study focuses solely on the perspectives of referring physicians regarding AI. Future research should compare these findings with the viewpoints of radiologists, patients, and other stakeholders to gain a more comprehensive understanding.

Another limitation of this study is the lack of assessment regarding physicians’ individual knowledge, familiarity, and practical experience with AI technologies. Although we collected data on participants’ overall years of clinical experience, we did not specifically measure their direct interaction with AI solutions within their respective subspecialties. Previous research suggests that exposure to and understanding of AI applications, as well as hands-on familiarity, can significantly influence levels of trust, acceptance, and perceived usefulness [17, 32]. Without capturing the degree of AI-specific knowledge and experience among participants, it remains unclear to what extent familiarity may have shaped their responses. Future studies should therefore include targeted measures of AI literacy and practical usage to better differentiate between skepticism arising from technical uncertainty and that based on broader ethical or systemic concerns. Such an approach would enable a more nuanced understanding of how experiential factors impact physicians’ attitudes towards AI in medical practice.

Lastly, this study does not fully account for broader socio-political and economic factors that may influence the integration of AI into clinical workflows. Elements such as national regulatory policies, reimbursement schemes, medico-legal uncertainties, and the degree of institutional support (e.g., infrastructure investments or training programs) are likely to significantly shape physicians’ willingness to adopt AI-based technologies. Future research should therefore incorporate macro-level variables to capture a more comprehensive picture of AI adoption dynamics in healthcare settings.

In conclusion, this study highlights physicians’ generally positive attitudes toward AI in radiology, especially for lesion detection and data analysis, which are seen as key to improving diagnostic accuracy. However, concerns about transparency, liability, and data privacy remain significant barriers. Addressing these issues through clear legal frameworks, better explainability of AI algorithms, and stronger data protection will be important for gaining trust among healthcare providers.

## Supplementary information

ELECTRONIC SUPPLEMENTARY MATERIAL

## Data Availability

The datasets generated and analyzed during the current study are available from the corresponding author upon reasonable request.

## Abbreviations

AI: Artificial intelligence
CT: Computed tomography

## References

[1] Hosny A, Parmar C, Quackenbush J, Schwartz LH, Aerts HJWL (2018) Artificial intelligence in radiology. Nat Rev Cancer 18:500–510. 10.1038/s41568-018-0016-5 29777175 10.1038/s41568-018-0016-5 PMC6268174

[2] Finck T, Moosbauer J, Probst M et al (2022) Faster and better: how anomaly detection can accelerate and improve reporting of head computed tomography. Diagnostics (Basel) 12:452. 10.3390/diagnostics12020452 35204543 10.3390/diagnostics12020452 PMC8871235

[3] Adamo SH, Gereke BJ, Shomstein S, Schmidt J (2021) From satisfaction of search to subsequent search misses: a review of multipletarget search errors across radiology and cognitive science. Cogn Res Princ Implic 6:59 10.1186/s41235-021-00318-w PMC8403090 34455466

[4] Ardila D, Kiraly AP, Bharadwaj S et al (2019) End-to-end lung cancer screening with three-dimensional deep learning on low-dose chest computed tomography. Nat Med 25:954–961. 10.1038/s41591-019-0447-x 31110349 10.1038/s41591-019-0447-x

[5] Ohlmann-Knafo S, Ramanauskas N, Jeyakumar EJ et al (2022) AI-based software for lung nodule detection in chest X-rays—time for a second reader approach? 10.48550/arXiv.2206.10912

[6] Cellina M, Cè M, Irmici G et al (2022) Artificial intelligence in emergency radiology: where are we going?. Diagnostics (Basel) 12:3223. 10.3390/diagnostics12123223 36553230 10.3390/diagnostics12123223 PMC9777804

[7] van Leeuwen KG, de Rooij M, Schalekamp S, van Ginneken B, Rutten MJCM (2022) How does artificial intelligence in radiology improve efficiency and health outcomes?. Pediatr Radiol 52:2087. 10.1007/s00247-021-05114-8 34117522 10.1007/s00247-021-05114-8 PMC9537124

[8] Lin L, Dou Q, Jin YM et al (2019) Deep learning for automated contouring of primary tumor volumes by MRI for nasopharyngeal carcinoma. Radiology 291:677–686. 10.1148/radiol.2019182012 30912722 10.1148/radiol.2019182012

[9] Tang Y, Zhang N, Wang Y et al (2022) Accurate and robust lesion RECIST diameter prediction and segmentation with transformers. 10.48550/arXiv.2208.13113

[10] Nalepa J, Kotowski K, Machura B et al (2022) Deep learning automates bidimensional and volumetric tumor burden measurement from MRI in pre- and post-operative glioblastoma patients. 10.48550/arXiv.2209.01402 10.1016/j.compbiomed.2023.106603 36738710

[11] Sosna J (2022) Deep learning for automated normal liver volume estimation. Radiology 302:343–344. 10.1148/radiol.2021212010 34698573 10.1148/radiol.2021212010

[12] Strohm L, Hehakaya C, Ranschaert ER, Boon WPC, Moors EHM (2020) Implementation of artificial intelligence (AI) applications in radiology: hindering and facilitating factors. Eur Radiol 30:5525–5532. 10.1007/s00330-020-06946-y 32458173 10.1007/s00330-020-06946-y PMC7476917

[13] Marey A, Arjmand P, Alerab ADS et al (2024) Explainability, transparency and black box challenges of AI in radiology: impact on patient care in cardiovascular radiology. Egyptian J Radiol Nucl Med 55:183. 10.1186/s43055-024-01356-2

[14] Marcus E, Teuwen J (2024) Artificial intelligence and explanation: how, why, and when to explain black boxes. Eur J Radiol 173:111393. 10.1016/j.ejrad.2024.111393 38417186 10.1016/j.ejrad.2024.111393

[15] Jha S, Topol EJ (2016) Adapting to artificial intelligence: radiologists and pathologists as information specialists. JAMA 316:2353–2354. 10.1001/jama.2016.17438 27898975 10.1001/jama.2016.17438

[16] Choudhury A, Asan O (2022) Impact of accountability, training, and human factors on the use of artificial intelligence in healthcare: exploring the perceptions of healthcare practitioners in the US. Human Fact Healthcare 2:100021. 10.1016/j.hfh.2022.100021

[17] Chen M, Zhang B, Cai Z et al (2022) Acceptance of clinical artificial intelligence among physicians and medical students: a systematic review with cross-sectional survey. Front Med 9:990604. 10.3389/fmed.2022.990604 10.3389/fmed.2022.990604 PMC9472134 36117979

[18] Lim SS, Phan TD, Law M et al (2022) Non-radiologist perception of the use of artificial intelligence (AI) in diagnostic medical imaging reports. J Med Imaging Radiat Oncol 66:1029–1034. 10.1111/1754-9485.13388 35191186 10.1111/1754-9485.13388 PMC10078783

[19] Huisman M, Ranschaert E, Parker W et al (2021) An international survey on AI in radiology in 1,041 radiologists and radiology residents part 1: fear of replacement, knowledge, and attitude. Eur Radiol 31:7058–7066. 10.1007/s00330-021-07781-5 33744991 10.1007/s00330-021-07781-5 PMC8379099

[20] Ferrario A, Loi M (2022) How explainability contributes to trust in AI. In: Proceedings of the 2022 ACM Conference on Fairness, Accountability, and Transparency, in FAccT ’22. Association for Computing Machinery, New York, NY, USA. pp. 1457–1466. 10.1145/3531146.3533202

[21] Jones C, Thornton J, Wyatt JC (2023) Artificial intelligence and clinical decision support: clinicians’ perspectives on trust, trustworthiness, and liability. Med Law Rev 31:501–520. 10.1093/medlaw/fwad013 37218368 10.1093/medlaw/fwad013 PMC10681355

[22] Mello MM, Guha N (2024) Understanding liability risk from using health care artificial intelligence tools. N Engl J Med 390:271–278. 10.1056/NEJMhle2308901 38231630 10.1056/NEJMhle2308901

[23] Radhwi OO, Khafaji MA (2024) The wizard of artificial intelligence: are physicians prepared?. J Fam Commun Med 31:344. 10.4103/jfcm.jfcm_144_24 10.4103/jfcm.jfcm_144_24 PMC11604183 39619463

[24] Contaldo MT, Pasceri G, Vignati G, Bracchi L, Triggiani S, Carrafiello G (2024) AI in Radiology: Navigating Medical Responsibility. Diagnostics (Basel) 14:1506 10.3390/diagnostics14141506 PMC11276428 39061643

[25] Marques M, Almeida A, Pereira H (2024) The medicine revolution through artificial intelligence: ethical challenges of machine learning algorithms in decision-making. Cureus 16:e69405. 10.7759/cureus.69405 39411643 10.7759/cureus.69405 PMC11473215

[26] Aggarwal N, Drew DA, Parikh RB, Guha S (2024) Ethical implications of artificial intelligence in gastroenterology: the co-pilot or the captain?. Dig Dis Sci 69:2727–2733. 10.1007/s10620-024-08557-9 39009918 10.1007/s10620-024-08557-9

[27] Bavli I, Ho A, Mahal R, McKeown MJ (2024) Ethical concerns around privacy and data security in AI health monitoring for Parkinson’s disease: insights from patients, family members, and healthcare professionals. AI & Soc. 10.1007/s00146-023-01843-6

[28] Marks M, Haupt CE (2023) AI Chatbots, health privacy, and challenges to HIPAA compliance. JAMA 330:309–310. 10.1001/jama.2023.9458 37410450 10.1001/jama.2023.9458

[29] Shah C, Nachand D, Wald C, Chen P-H (2023) Keeping patient data secure in the age of radiology artificial intelligence: cybersecurity considerations and future directions. J Am Coll Radiol 20:828–835. 10.1016/j.jacr.2023.06.023 37488026 10.1016/j.jacr.2023.06.023

[30] Kelly BS, Quinn C, Belton N, Lawlor A, Killeen RP, Burrell J (2023) Cybersecurity considerations for radiology departments involved with artificial intelligence. Eur Radiol 33:8833–8841. 10.1007/s00330-023-09860 37418025 10.1007/s00330-023-09860-1 PMC10667413

[31] Hasani N, Morris MA, Rhamim A et al (2022) Trustworthy artificial intelligence in medical imaging. PET Clin 17:1. 10.1016/j.cpet.2021.09.007 34809860 10.1016/j.cpet.2021.09.007 PMC8785402

[32] Kauttonen J, Rousi R, Alamäki A (2025) Trust and acceptance challenges in the adoption of AI applications in health care: quantitative survey analysis. J Med Intern Res 27:e65567. 10.2196/65567 10.2196/65567 PMC11971584 40116853

