# Supplementary material for: Implementation of AI in radiology: the perspective of referring physicians
Source: Insights Imaging. 2025 Oct 31;16:238. doi: 10.1186/s13244-025-02120-4 (PMC12579084; doi:10.1186/s13244-025-02120-4)
Supplement: Supplementary file 1 — ELECTRONIC SUPPLEMENTARY MATERIAL [file 13244_2025_2120_MOESM1_ESM.pdf]

Implementation of artificial intelligence (AI) in radiology: The Perspective of Referring Physicians

ELECTRONIC SUPPLEMENTARY MATERIAL

Supplementary Figure 1: Questionnaire

Implementation of artificial intelligence (AI) in radiology

What field do you work in?

Please choose...

How many years of professional experience do you have?

#

0 / 80

Please select your field of activity.

- ○ Practice doctor
- ○ Resident physician in a clinic
- ○ Senior physician in a clinic
- ○ Chief physician in a clinic

How do you rate the application of artificial intelligence in radiological diagnosis?

This means AI support for radiologists in identifying pathologies. The radiologist is responsible for the final interpretation of the pathologies found.

Very negative — Very positive

1	2	3	4	5

How could trust in the application of artificial intelligence in radiological diagnostics be improved?

- ◯ Transparency of the AI model (explainability of how it works, disclosure of the data set used to train the AI model)
- ◯ Responsibility and liability (clarification of the liability question, who is liable in the event of damage? Doctor or AI provider?
- ◯ Data protection audits (mechanisms for regularly checking data protection measures and monitoring compliance.)
- ◯ other

Please rate the importance of different application areas of artificial intelligence (AI) in radiology. Assign a priority to the following areas, with 1 being the highest priority and 6 being the lowest priority?

1 = the highest priority
6 = the lowest priority

- Lesion detection (AI models can detect and mark lesions such as tumors or vascular abnormalities)
- Automated determination of organ volumes
- Workflow management for radiologists through prioritization of image data sets to be evaluated.

  (Image data sets are sorted according to their urgency and clinical relevance so that critical cases can be addressed early and routine cases can be prioritized accordingly.)
- Automated tumor volume determination

  (so far tumors have only been measured manually in 3 levels)
- Automated image quality control

  (Medical image data is reviewed for its technical quality. Parameters such as image sharpness, signal-to-noise ratio, artifacts, and correct patient positioning are analyzed. The goal is to detect images of inadequate quality at an early stage to avoid repeat examinations.)

1.
